# Terahertz Transmission through a Gold Mirror or Electrode

**DOI:** 10.3390/ma17163942

**Published:** 2024-08-08

**Authors:** Fabio Novelli

**Affiliations:** Department of Physical Chemistry II, Ruhr University Bochum, 44801 Bochum, Germany; fabio.novelli@rub.de

**Keywords:** terahertz, gold, chromium, metal, film, borosilicate, conductivity, resistivity

## Abstract

Hundreds of nanometer-thick metal layers are used as electrical conductors in various technologies and research fields. The intensity of the radiation transmitted by such devices is a small fraction and is often neglected. Here, it is shown that intense terahertz time-domain spectroscopy can probe the absolute electro-optical properties of a 100 nm thick gold sample in transmission geometry without the need to apply electrical contacts or handle wires. The terahertz conductivity of the metal film agrees with that obtained from standard contact measurements of the static component within the error bars. This experimental approach can help to quantify the electrical properties of opaque and conductive materials such as the composite electrodes used in photovoltaic or electrochemical applications, and in the quality control of metal films.

## 1. Introduction

Gold is used in countless applications spanning basic research and technology development. Owing to their high reflectivity and excellent electrical conductivity, gold films with thicknesses on the order of 100 nm are routinely used as mirrors [1,2,3,4,5] or electrodes in solar light harvesting [6,7,8] and electrochemical devices [9,10]. In optical experiments, owing to the minimal amount of radiation transmitted, the properties of gold films are often studied in reflection geometry. However, reflection measurements are strongly influenced by the outermost layer, with limited information about deeper portions of the sample. In electrochemical experiments, the overall performance of a device can be studied as a function of the properties of the electrode, such as its thickness or morphology [11,12,13]. Four-point probe (4PP) measurements are routinely used to estimate the electrical conductivity of the sole electrode. However, 4PP requires electrical wiring and contacts and can only estimate the real part of the conductivity close to zero frequency.

In this context, terahertz time-domain spectroscopy (THz-TDS) complements 4PP. THz-TDS can measure the electro-optical conductivity of a sample at terahertz frequencies, not only the static component, and without applying electrodes; i.e., it is a contactless technique. In previous work, Naftaly et al. [14] found that different techniques agreed in the estimation of the sheet resistance of transparent thin films made of Indium Gallium Zinc Oxide. Here, this approach is extended to highly conductive metal samples such as 100 nm thick gold layers: a bright radiation source can be used to measure the transmission of an opaque material [15,16,17,18], as was previously demonstrated on aqueous solutions [19].

Only a handful of previous works have addressed the properties of thick gold films with THz-TDS, either in transmission or reflection geometries or with the help of waveguides [20,21,22]. Laman and Grischkowsky [20] estimated the real part of the conductivity of 85 and 150 nm thick gold samples averaged between ~0.5 and 3.5 THz. No error bars were given, and no comparison with standard conductivity measurements was reported. Here, with the aid of intense terahertz time-domain spectroscopy (iTHz-TDS), the optical properties and conductivity of a 100 nm thick gold mirror were obtained. To this end, the dielectric functions of the bare glass substrate and the adhesion layer of chromium were addressed independently. Statistical error bars are provided for all the materials studied. The electrical conductivity of the gold mirror at frequencies between 0.3 and 1.1 THz agreed with the static conductivity value obtained from contact electric measurements within the error bars. This work paves the way for the contactless electro-optical characterization of highly conductive, opaque, and composite electrodes.

## 2. Materials and Methods

The experimental setup is similar to the one shown in Figure 5 of ref. [23]. The main difference is the position of the sample, which is not at the focal point. Additional experimental details are provided in ref. [19]. In short, intense terahertz radiation is generated by the tilted-front optical rectification of an amplified laser pulse, with a central wavelength of ~800 nm and a ~100 fs pulse duration, in a lithium niobate crystal. The generated THz power, measured by a THZ12D-3S-VP-D0 detector (Gentec-EO, Quebec City, QC, Canada), amounted to approximately 0.5 mW. To avoid possible non-linear effects [24,25,26,27,28,29], the specimen was placed away from the focus, in a position where the radius of the THz beam was 10 mm, resulting in peak fields of about 10 kV/cm. The transient, oscillating fields were detected by delaying a near-infrared electro-optical sampling pulse that overlapped with the THz beam in a 0.5 mm thick gallium phosphide (GaP) crystal. Each THz pulse was detected over a temporal range of 8.2 ps in 0.1 ps steps by scanning a mechanical delay stage. It was empirically found that the frequency range over which the terahertz spectrometer displays the best signal-to-noise ratio is between about 0.3 and 1.1 THz. For this reason, the experimental results reported here are shown over this frequency range. The relative humidity was reduced by purging the spectrometer chamber with nitrogen gas and remained at (9 ± 1)% during all the measurements.

The sample, 10-AU8633-1, was bought from Micro to Nano BV (Haarlem, Netherlands). Schematics of the sample are available on the manufacturer’s website. It is made of 100 nm gold deposited onto a 5 nm thick adhesion layer of chromium on top of a 1 mm thick borosilicate glass. The manufacturer indicated a thickness tolerance of 5% and provided the static resistivity (conductivity) of the sample, $\rho_{DC}\approx 3.45\cdot 10^{-6}\;\Omega \cdot cm$ ($1/\rho_{DC}=\sigma_{DC}\approx 2.9\cdot 10^{6}\;\Omega^{-1}{cm}^{-1}$), which was measured with a 4PP method. The sample was mounted in an aluminum holder that, in turn, was magnetically attached to a copper plate whose temperature was stabilized by a chiller to 20.0 ± 0.1 °C. As reported previously [26], the gold layer was removed from a part of the sample by soaking in aqua regia. The chromium film—which appears gray to the eye—was further removed on a smaller portion of the specimen by mechanical polishing. In this way, the sample was divided into three regions: one with the multiple stacked layers of gold, chromium, and glass; one with the thin chromium film on top of the borosilicate substrate; and one where only glass is present.

## 3. Results

**Figure 1:**
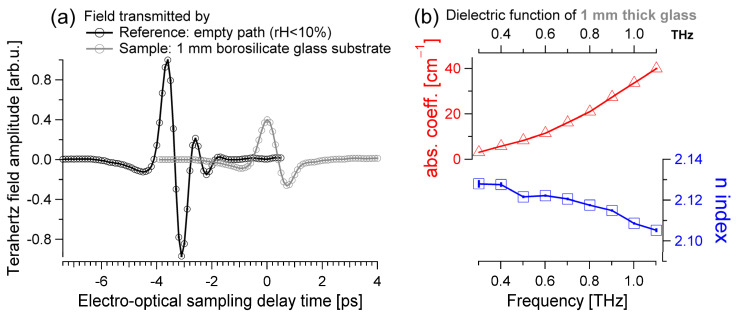
(**a**) Terahertz field transmitted by the empty path (black) and by the 1 mm thick glass substrate (gray). The field transmitted by the glass sample is delayed in time by about 3.5 ps and reduced in amplitude. (**b**) The absorption coefficient (red) and the index of refraction (blue) of the glass coverslip are estimated by assuming an optically thick material and high-absorption/low-refraction approximation. The errors are equal to the standard deviation calculated from 25 independent measurements of both sample and reference THz fields. The errors are shown with vertical bars for each data point and are often too small to be visible, i.e., they can be smaller than the line thickness.

The time-dependent THz fields transmitted by the empty path (without the sample) and by the bare glass substrate are shown with the black and gray traces in Figure 1a, respectively. Each trace corresponds to the average of 25 consecutive scans of one THz field, resulting in a total measurement time of about 5 min for each sample. The error bars represent the standard deviation (SD) calculated from 25 independent measurements of the transmitted THz pulse. As is customary in terahertz time-domain spectroscopy, the optical properties of a material can be estimated by comparing the phase-resolved and frequency-dependent fields transmitted by a sample, $E_{sam}\left(\omega \right)$, with the ones transmitted by a reference, $E_{ref}\left(\omega \right)$, where $\omega /\left(2\pi \right)=\upsilon$ is the frequency. Each one of those complex and frequency-dependent functions, $E_{sam}\left(\omega \right)$ and $E_{ref}\left(\omega \right)$, can be obtained by Fourier transformation (FT) of the corresponding THz field detected in the time domain, e.g., the black and gray traces in Figure 1a. To estimate the dielectric function of the borosilicate glass coverslip sample ($E_{sam}\left(\omega \right)=E_{glass}\left(\omega \right)$), a reference of nitrogen-purged air is recorded too ($E_{ref}\left(\omega \right)=E_{air}\left(\omega \right)$). The glass sample is optically thick: the THz pulses that originate from reflections at the glass/air interfaces are delayed beyond the detection time window and can be ignored. As can be seen in Figure 1a, the first THz pulse transmitted by the 1 mm thick borosilicate substrate (gray in Figure 1a) was delayed by about +3.5 ps with respect to the peak THz field transmitted by an equal thickness of air (black in Figure 1a). The subsequent, further delayed THz pulse stems from two reflections at the glass/air interfaces and travels through glass two more times, accumulating ca. 3.5 ps × 2 ~ +7 ps of delay with respect to the gray field in Figure 1a. As the time acquisition window varies from about −4 ps to +4 ps, the etalons due to the Fabry–Pérot effect can be ignored (+7 ps ≫ +4 ps). For optically thick materials, which do not absorb strongly at THz frequencies, the real part of the index of refraction, $n\left(\omega \right)$, and the absorption coefficient, $\alpha \left(\omega \right)$, can be estimated with the following equations [30]:

(1) $$n_{glass}\left(\omega \right)=1+\frac{c}{\omega d}arg\left(\frac{E_{glass}\left(\omega \right)}{E_{air}\left(\omega \right)}\right)$$

(2) $$\alpha_{glass}\left(\omega \right)=-\frac{2}{d}\ln\left(\left|\frac{E_{glass}\left(\omega \right)}{E_{air}\left(\omega \right)}\right|\right)-\frac{2}{d}\ln\left(\frac{{\left(n_{glass}+1\right)}^{2}}{4\cdot n_{glass}}\right)$$

where $arg\left(\frac{E_{glass}\left(\omega \right)}{E_{air}\left(\omega \right)}\right)$ is the phase difference and $\left|\frac{E_{glass}\left(\omega \right)}{E_{air}\left(\omega \right)}\right|$ the magnitude ratio of the FT fields transmitted by the glass sample and the air reference, respectively. The sample thickness is equal to d and c is the speed of light. The optical properties of the borosilicate glass obtained with Equations (1) and (2) are reported in Figure 1b. The index of refraction has a weak dependence on the THz wavelength and has a value close to 2.1. The absorption coefficient increases almost exponentially at higher probe frequencies and reaches a value of ~30 cm^−1^ at 1 THz. These results agree with previous reports [31,32,33].

**Figure 2:**
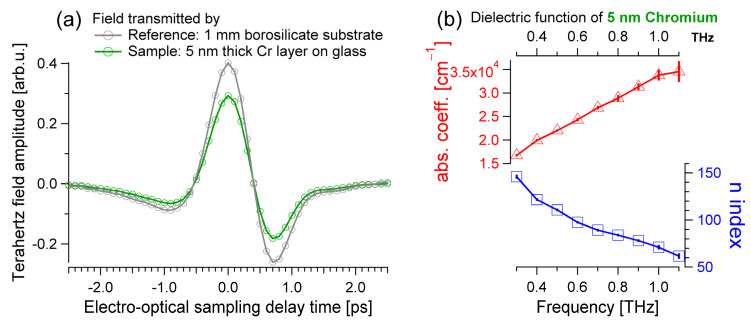
(**a**) Terahertz field transmitted by the bare borosilicate glass substrate reference (gray) and by the sample composed of a 5 nm thick chromium film deposited on the glass (green). The sample is too thin to induce a noticeable time delay of the THz pulse. The transmitted amplitude is smaller for the film. (**b**) The absorption coefficient (red) and index of refraction (blue) of the chromium layer are estimated by assuming an optically thin material and the thin-film approximation by Tinkham.

The dielectric function of the thin chromium film can be obtained from the THz fields transmitted by the bare glass substrate (gray in Figure 1a and Figure 2a), and by the transmission of the chromium-on-glass sample, which is the green THz trace in Figure 2a. A material is optically thin if the multiple reflections have a large enough amplitude to be detected and cannot be separated in time from the main pulse. In the simplest terms, electromagnetic radiation acquires a temporal delay t≈d·n/c by traversing a material of thickness d and index of refraction n. The additional delay accumulated by the first Fabry–Pérot term corresponds to [19] ∆t≈2·d·n/c. As the chromium layer has a thickness d=5 nm and an index of refraction that is roughly close to [34,35] 100, ∆t≈3 fs. This retardation is much smaller than the duration of the laser pulse (~100 fs). Thus, multiple reflections are part of the THz signal transmitted by the chromium film (green trace in Figure 2a). It is possible to obtain analytical equations for the optical functions of a thin slab of a material within the thin-film or Tinkham’s approximation, which is valid if ω·d·n/c is much smaller than 1. This holds in the case of the chromium film, for which ω·d·n/c≈0.01≪1 at a 1 THz probe frequency. Thus, the Tinkham formula can be used in this case [36,37,38,39,40,41]:

(3) $$\sigma \left(\omega \right)=\frac{1}{Z_{0}d}\left(1+n_{glass}\left(\omega \right)+i\frac{c}{2\omega }\alpha_{glass}\left(\omega \right)\right)\frac{E_{glass}\left(\omega \right)-E_{Cr}\left(\omega \right)}{E_{Cr}\left(\omega \right)}$$

where $\sigma \left(\omega \right)=\sigma_{1}\left(\omega \right)+i\sigma_{2}\left(\omega \right)$ is the complex and frequency-dependent conductivity that includes real ($\sigma_{1}$) and imaginary ($\sigma_{2}$) terms, i is the imaginary unit, $Z_{0}\approx 376.7\;\Omega$ is the constant impedance of free space, $n_{glass}\left(\omega \right)$ and $\alpha_{glass}\left(\omega \right)$ are the optical properties of the bare glass substrate displayed in Figure 1b, and $E_{Cr}\left(\omega \right)$ is the complex FT of the terahertz field transmitted through the thin chromium sample layer on top of its substrate glass material, i.e., the FT of the green trace in Figure 2a. From $\sigma_{1}\left(\omega \right)$ and $\sigma_{2}\left(\omega \right)$, it is possible to estimate all the other optical functions [42]. The real part of the dielectric function is $\varepsilon_{1}\left(\omega \right)=1-\sigma_{2}\left(\omega \right)/\left(\epsilon_{0}\omega \right)$ and the imaginary component is $\varepsilon_{2}\left(\omega \right)=\sigma_{1}\left(\omega \right)/\left(\epsilon_{0}\omega \right)$, with $\epsilon_{0}\approx 88.54\;fs/\left(\Omega \cdot \mathrm{cm}\right)$ vacuum permittivity constant. The real part of the index of refraction can be calculated from the dielectric function, $n\left(\omega \right)=\sqrt{+\varepsilon_{1}\left(\omega \right)+\sqrt{\varepsilon_{1}^{2}\left(\omega \right)+\varepsilon_{2}^{2}\left(\omega \right)}}/\sqrt{2}$, and the absorption is $\alpha \left(\omega \right)=2\omega k\left(\omega \right)/c=2\omega \sqrt{-\varepsilon_{1}\left(\omega \right)+\sqrt{\varepsilon_{1}^{2}\left(\omega \right)+\varepsilon_{2}^{2}\left(\omega \right)}}/\left(\sqrt{2}c\right)$, with $k\left(\omega \right)$ extinction coefficient. In keeping with the results shown for the borosilicate glass in Figure 1, the index of refraction and the absorption coefficient of the thin chromium film are shown in Figure 2b. These results are similar to the ones published previously [34,35].

**Figure 3:**
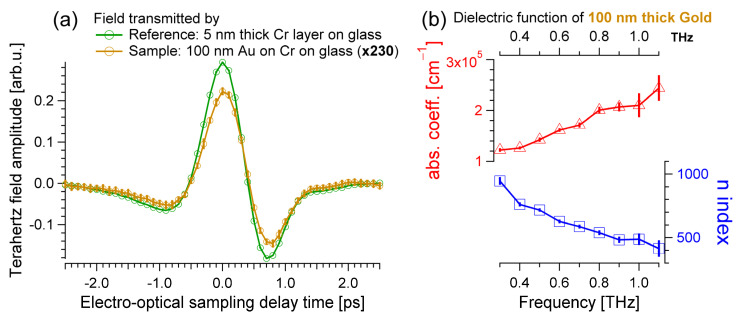
(**a**) Terahertz field transmitted by the chromium-on-glass reference (green) and by the sample composed of the multi-layered gold/chromium/borosilicate sample (orange). The transmitted amplitude is much smaller for the gold mirror, which is multiplied by 230 for display purposes. Error bars on the order of ±5% are visible for the golden specimen. (**b**) As detailed in the text, the absorption coefficient (red) and the index of refraction (blue) of gold can be estimated numerically.

The dielectric function of gold can be obtained from the pulsed THz fields transmitted by the chromium-on-glass reference (green in Figure 2a and Figure 3a) and by the transmission of the full, multilayered metal mirror sample encompassing gold, chromium, and glass, which is shown with the orange THz trace in Figure 3a. Please note that the amplitude of the THz field transmitted by the gold mirror is very small. For this reason, and for display purposes only, the orange curve is multiplied by a factor 230 in Figure 3a. For a gold layer, it is not possible to adopt the previous approximation of an optically thick and low absorbing material used for the glass (Figure 1), nor the thin film Tinkham formula used for the chromium sample (Figure 2). A 100 nm thick gold mirror is optically thin, i.e., the Fabry–Pérot etalons fall within the acquisition time window (∆t≈2·d·n/c≈0.5 ps for d=100 nm and n≈750), but the thin film approximation cannot be used because ω·d·n/c≈2π·1 THz·100 nm·750/c≈1.6 is larger than 1. To estimate the optical properties of gold, the stacked sample geometry must be considered fully, and the resulting complex equation solved numerically. The transmission of a composite sample made of an optically thick layer of nitrogen-purged air, optically thin gold, optically thin chromium, optically thick glass, and optically thick air is as follows [19]:

(4) $$E_{Au}\left(\omega \right)=E_{0}\left(\omega \right)\frac{t_{air/Au}\cdot t_{Au/Cr}\cdot t_{Cr/glass}\cdot t_{glass/air}\cdot \varphi_{Au}\cdot \varphi_{Cr}\cdot \varphi_{glass}}{\left(1-\varphi_{Au}^{2}\cdot r_{Au/Cr}\cdot r_{Au/air}\right)\left(1-\varphi_{Cr}^{2}\cdot r_{Cr/Au}\cdot r_{Cr/glass}\right)}$$

where $E_{0}\left(\omega \right)$ is the input THz field emitted by the source and impinging on the sample, $t_{p/q}$ ($r_{p/q}$) are the transmission (reflection) Fresnel coefficients at the interface between medium p and medium q, and $\varphi_{s}=e^{i\frac{\omega }{c}d_{s}n_{s}\left(\omega \right)}e^{-d_{s}\alpha_{s}\left(\omega \right)/2}$ is the complex phase acquired by an electromagnetic pulse that has traveled through the thickness $d_{s}$ of the s sample, which has refraction and absorption coefficients equal to $n_{s}\left(\omega \right)$ and $\alpha_{s}\left(\omega \right)$, respectively. Each Fresnel term depends on the indexes of refraction and absorption (or extinction) coefficients of the two materials forming the interface [43]. The reference field is transmitted by the air/chromium/glass/air sandwich and can be written as

(5) $$E_{Cr}\left(\omega \right)=E_{0}\left(\omega \right)\frac{t_{air/Cr}\cdot t_{Cr/glass}\cdot t_{glass/air}\cdot \varphi_{air}\cdot \varphi_{Cr}\cdot \varphi_{glass}}{1-\varphi_{Cr}^{2}\cdot r_{Cr/glass}\cdot r_{Cr/air}}$$

with $\varphi_{air}$ phase acquired by an electromagnetic pulse that propagated through an air layer as thick as the gold film: 100 nm. Equations (4) and (5) are obtained by assuming the convergence of the geometric series describing the etalons, i.e., that $\sum_{k=0}^{\infty }x^{k}=1/\left(1-x\right)$, which is valid for $\left|x\right|<1$. Here, the term x is either $\varphi_{Au}^{2}r_{Au/Cr}r_{Au/air}$, $\varphi_{Cr}^{2}r_{Cr/Au}r_{Cr/glass}$ or $\varphi_{Cr}^{2}r_{Cr/glass}r_{Cr/air}$. For example, convergence is warranted if all the materials involved have positive indexes of refraction and absorption coefficients, which is also the case here. The complex and frequency-dependent ratio of Equations (4) and (5) is

(6) $$\frac{E_{Au}\left(\omega \right)}{E_{Cr}\left(\omega \right)}=\frac{t_{air/Au}\cdot t_{Au/Cr}\cdot \varphi_{Au}\;}{t_{air/Cr}\cdot \varphi_{air}}\frac{1-\varphi_{Cr}^{2}\cdot r_{Cr/glass}\cdot r_{Cr/air}}{\left(1-\varphi_{Au}^{2}\cdot r_{Au/Cr}\cdot r_{Au/air}\right)\left(1-\varphi_{Cr}^{2}\cdot r_{Cr/Au}\cdot r_{Cr/glass}\right)}$$

which includes the input parameters of the refraction and the absorption of both the glass substrate (Figure 1b) and the chromium film (Figure 2b). The dielectric function of the 100 nm thick gold sample can be obtained by solving Equation (6) numerically. The THz fields transmitted by sample and reference, the orange and green pulses in Figure 3a, are FT, and their ratio is calculated. Such an experimentally determined quantity corresponds to the left side of Equation (6). This experimental complex number is equated to the right side of Equation (6), at each THz frequency, which is a complex-valued mathematical expression depending on the index of refraction and the absorption coefficient of gold and on the parameters that were determined previously, as shown in Figure 1b and Figure 2b. The results of this numerical analysis, performed under the approximation of normal incidence, $n_{air}\left(\omega \right)=1$, and $k_{air}\left(\omega \right)=0$, are displayed in Figure 3b. The vertical error bars are the SD obtained from the 25 pairs of THz fields recorded for the sample (air/Au/Cr/glass/air) and reference (air/Cr/glass/air). The index of refraction of the 100 nm gold mirror is found within ca. 500 and 1000 at frequencies between 0.3 THz and 1.1 THz, while the absorption coefficient varies between ~100 k cm^−1^ and ~250 k cm^−1^ over the same spectroscopic range, resulting in penetration depths spanning from about 100 nm to 40 nm.

## 4. Discussion

The optical properties of gold in the dielectric and infrared ranges can be approximated to the free electron gas or Drude model [44,45,46,47,48,49,50], whereby the complex and frequency-dependent optical conductivity is $\sigma \left(\omega \right)=\sigma_{DC}/\left(1-i\omega \tau \right)$ with τ carrier scattering time and $\sigma_{DC}$ static conductivity. The static or DC conductivity is equal to $\sigma_{DC}=\varepsilon_{0}\omega_{P}^{2}\tau$, where $\omega_{P}/\left(2\pi \right)=\nu_{P}$ is the plasma frequency. The room temperature DC conductivity of bulk gold is equal to [51] $\sigma_{DC}=4.52\cdot 10^{6}\;\Omega^{-1}{cm}^{-1}$. This corresponds to the plasma frequency [37] $\nu_{P}\sim 2230\;THz$ and the scattering time τ~26 fs. For thicknesses between about 10 and 50 nm, previous reports indicated smaller plasma frequencies (from $\nu_{P}\sim 1980\;THz$ to $\nu_{P}\sim 2140\;THz$) and scattering times (τ~15−22 fs) [22,39,50,52].

**Figure 4:**
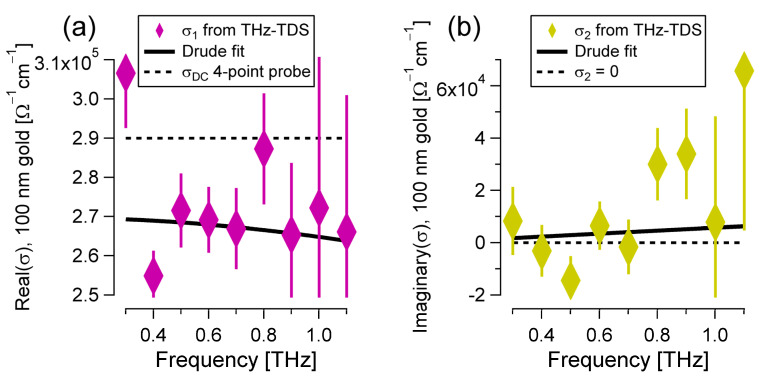
(**a**) Real and (**b**) imaginary parts of the optical conductivity of the 100 nm thick gold mirror. These quantities, together with the error bars corresponding to plus-or-minus one standard deviation, are estimated numerically from Equation (6), the measured THz fields shown in Figure 3a, and the optical properties of the glass substrate and the thin chromium adhesion layer. The solid black lines are the results of a global Drude fit, and the dashed ones are shown for comparison.

Along with the refractive and absorptive properties of the 100 nm thick gold mirror reported in Figure 3b, the electrical conductivity at THz frequencies can be estimated as well. For example [42], $\sigma_{1}\left(\omega \right)=\epsilon_{0}c\cdot n\left(\omega \right)\cdot \alpha \left(\omega \right)$ and $\sigma_{2}\left(\omega \right)=\omega \epsilon_{0}\left[1-n^{2}\left(\omega \right)+k^{2}\left(\omega \right)\right]$. The purple diamonds in Figure 4a display the real part of the optical conductivity of the gold films. Figure 4b reports the imaginary component of the dielectric conductivity, in yellow. The error bars are obtained directly from the 25× repeated measurements and correspond to ±1 SD. The value of $\sigma_{1}\left(\omega \right)$ agrees reasonably well, within one or two error bars, with the value obtained with the 4PP measurement, which is shown with the dashed black line in Figure 4a. As expected, the value of $\sigma_{2}\left(\omega \right)$ is close to zero [38], which is marked with a dashed black line in Figure 4b. The real and imaginary conductivities of the gold film can be simultaneously fit to the two functions describing the Drude model, $\sigma_{1}\left(\omega \right)=\sigma_{DC}/\left(1+\omega^{2}\tau^{2}\right)$ and $\sigma_{2}\left(\omega \right)=\omega \tau \sigma_{DC}/\left(1+\omega^{2}\tau^{2}\right)$. The results are shown with solid black curves in Figure 4a,b. The best values and error bars obtained from the fit are $\nu_{P}=1887\pm 372\;THz$ and τ=22±9 fs, which are consistent with the bulk values [37] ($\nu_{P}\sim 2230\;THz$, τ~26 fs) and previous studies on thin gold films ($\nu_{P}\sim 1980-2140\;THz$, τ~15−22 fs) [22,39,50,52]. By looking at the spread of the data points in Figure 4a, the static conductivity is estimated to $\sigma_{DC}=\left(2.7\pm 0.2\right)\cdot 10^{6}\;\Omega^{-1}{cm}^{-1}$, which agrees with the 4PP measurement ($\sigma_{DC}\approx 2.9\cdot 10^{6}\;\Omega^{-1}{cm}^{-1}$).

## 5. Conclusions

This work demonstrates that it is possible to correctly quantify the optical conductivity of 100 nm thick gold films—a common thickness for electrodes or mirrors—with intense terahertz time-domain spectroscopy (iTHz-TDS) in transmission geometry. This approach is complementary to standard electrical measurements, which only probe static conductivity, and to reflection experiments, which are intrinsically more sensitive to the sample layers that are closer to the interface.

The dielectric functions of the 1 mm thick bare borosilicate substrate, of the 5 nm thin chromium film, and of the 100 nm gold layer were disentangled with a series of independent experiments, as detailed in Figure 1, Figure 2, and Figure 3, respectively. Appropriate data analysis was used for each sample: the glass is optically thick and absorbs the terahertz radiation weakly; the chromium is a thin film that can be described with the Tinkham formula; and the response of the gold layer must be analyzed using a numerical approach. The estimated optical conductivity and the Drude-fitted parameters of the gold mirror qualitatively agree with previous reports and, importantly, with the static conductivity independently estimated using electrical contacts.

As iTHz-TDS is intrinsically a contactless technique, these results are particularly important for the characterization of opaque electrical conductors that are difficult to measure by applying electrical contacts and wires. This includes the porous and composite electrodes used, for example, in electro-chemical processes [53,54] or solar light harvesting and photovoltaics [55,56,57,58,59]. As terahertz reflections are currently used to monitor the deposition of paint in the automotive industry [60], other possible applications of iTHz-TDS could include the quality control of metal films, which are used in various technological and research fields spanning both electronics and optics.

## Data Availability

The original contributions presented in the study are included in the article, and further inquiries can be directed to the corresponding author.
